# Effects of Matrix Composition and Temperature on Viability and Metabolic Activity of Microencapsulated Marine Bacteria

**DOI:** 10.3390/microorganisms10050996

**Published:** 2022-05-10

**Authors:** Emily Pope, Bradley Haltli, Russell G. Kerr, Ali Ahmadi

**Affiliations:** 1 Department of Biomedical Science, Atlantic Veterinary College, University of Prince Edward Island, Charlottetown, PE C1A 4P3, Canada; epope4355@upei.ca (E.P.); bhalti@upei.ca (B.H.); rkerr@upei.ca (R.G.K.); 2 Nautilus Biosciences Croda, Charlottetown, PE C1A 4P3, Canada; 3 Department of Chemistry, University of Prince Edward Island, Charlottetown, PE C1A 4P3, Canada; 4 Faculty of Sustainable Design Engineering, University of Prince Edward Island, Charlottetown, PE C1A 4P3, Canada; 5 Department of Mechanical Engineering, École de Technologie Supérieure (ÉTS), Montreal, QC H3C 1K3, Canada

**Keywords:** natural product discovery, bacteria cultivation, uncultivable bacteria, microencapsulation

## Abstract

To enhance the discovery of novel natural products, various innovations have been developed to aid in the cultivation of previously unculturable microbial species. One approach involving the microencapsulation of bacteria has been gaining popularity as a new cultivation technique, with promising applications. Previous studies demonstrated the success of bacterial encapsulation; however, they highlighted that a key limitation of encapsulating bacteria within agarose is the high temperature required for encapsulation. Encapsulation of bacteria within agarose typically requires a temperature high enough to maintain the flow of agarose through microfluidic devices without premature gelation. Given the sensitivity of many bacterial taxa to temperature, the effect of various agarose-based encapsulating matrices on marine bacterial viability was assessed to further develop this approach to bacterial culture. It was determined that lowering the temperature of encapsulation via the use of low-gelling-temperature agarose, as well as the addition of nutrients to the matrix, significantly improved the viability of representative marine sediment bacteria in terms of abundance and metabolic activity. Based on these findings, the use of low-gelling-temperature agarose with supplemental nutrients is recommended for the encapsulation of marine bacteria obtained from temperate habitats.

## 1. Introduction

A key issue in the discovery of modern microbial natural products is the inability to culture the vast majority of the microbial world [1,2]. This critical issue has been termed the ”Great Plate Count Anomaly” since it is known that the growth of microbial colonies observed within the laboratory does not accurately represent the true microbial biodiversity [3]. The ability to culture microbes is vital for gaining a greater understanding of these organisms and the unique compounds they produce, which have potential pharmaceutical and biotechnological applications [4]. As a result, various methods have been employed over the years in an attempt to culture a greater proportion of microbes from various environments. The simplest of these methods includes the modification of growth characteristics and media components to facilitate the growth of different species [5]. Another technique, known as co-culture, allows for the transmission of chemical signals between organisms as some microbes are incapable of growing without specific signal molecules from nearby helper bacteria [1,6]. The incubation of bacterial cells within their natural habitat, a technique known as in situ incubation, is a cultivation strategy that eliminates the need to optimize various parameters within the laboratory setting, including, but not limited to, temperature, salinity, pH, nutrient content, and aeration [1,6]. More recently, the use of single-cell isolation in combination with miniaturization has led to a more high-throughput approach to the discovery of individual species, as miniaturization allows for the testing of increased sample sizes, and the initial separation of individual species allows for a more streamlined downstream processing [7]. Furthermore, the combination of these various techniques is expected to enable the cultivation of an even greater proportion of microbes [1].

Microencapsulation of bacteria represents a relatively new cultivation technique that overcomes some key limitations in previous culturing techniques through the combination of previous principles [7]. The encapsulation of bacteria within agarose to form microbeads acts as a form of dilution to extinction, which may aid slower-growing bacteria by preventing competition among organisms each confined to their own microbead when single-cell encapsulation is achieved [8]. Additionally, the formation of microbeads via microencapsulation acts as a form of miniaturization that allows for the processing of a larger sample size thus facilitating greater throughput. Microbeads can also be incubated in a particular environment, which allows all environmental parameters to be maintained while undergoing in situ incubation, increasing the likelihood that cultivated cells will survive the transition to a laboratory setting [9]. The use of a nutrient permeable matrix such as agarose to separate individual cells still allows for a form of co-culture as well since cell signals may be transmitted between cultures in adjacent beads [10]. Thus, the combination of microencapsulation with in situ incubation encompasses all previous methods into one method. Our previous research has demonstrated that encapsulation in agarose is well-suited to mesophilic bacteria; however, it may adversely affect the viability of psychrophilic bacteria due to the relatively high temperature required for encapsulation [11]. 

While various gelling biomaterials may be used for the encapsulating matrix, including agarose, alginate, gellan gum, etc., agarose may represent the most versatile and compatible matrix due to its stability, biocompatibility, and ease of preparation [12]. Agarose allows for nutrient diffusion into the microbead from the surrounding environment, allowing the physical and chemical environment surrounding the cells to mimic the natural habitat when microbeads are incubated in situ [13]. Additionally, the use of agarose allows for future adaptations of the type of agarose utilized to form the microbeads depending on the goal of the study; for instance, if working in a cooler environment where heat stress may be detrimental to certain species, agarose with low gelling temperature (35 °C) can be used in place of traditional ultrapure agarose (45 °C). Alternatively, if isolating microbes from a eutrophic environment, nutrients can be added to the encapsulation matrix, or if attempting to culture a specific taxa of bacteria, such as Actinobacteria, the agarose can be amended with specific nutrients to aid in selective isolation of targeted taxa for this purpose. 

Different growth requirements, including temperature tolerance, salinity requirement, nutrient requirement, and motility, along with various other factors, contribute to the viability of species following encapsulation. Thus, it is expected that the use of different encapsulating matrices to support different growth requirements will be beneficial in the cultivation of species that have previously been difficult to cultivate within a laboratory setting. For instance, various matrices such as agarose, collagen, chitosan, alginate, gelatin, etc. have been used in different studies to encapsulate bacteria [10,11]. More specifically, these matrices may be modified further to select for the growth and survival of specific organisms. For instance, previous studies have shown that agarose with low gelling temperature benefits slow-growing bacteria and that it may also improve the viability of psychrophilic bacteria [14]. Consequently, the use of agarose with low gelling temperature may decrease the detrimental impacts of high encapsulating temperatures required for standard agarose while preventing premature gelation of microbeads during the formation process. As most bacteria require some source of nutrients to survive, the addition of nutrients to the matrix may also improve bacterial viability.

As the effect of matrix composition on the microencapsulation of marine bacteria has not been previously investigated, the viability of three representative marine bacterial species previously isolated by us from our local temperate marine environment, Prince Edward Island, Canada (*Marinomonas polaris*, *Psychrobacter aquimaris*, and *Bacillus licheniformis*) was investigated using four different agarose-based matrices [11,15]. Some of the characteristics of the representative bacteria utilized for encapsulation are summarized in Table 1. The impact of modifying the encapsulating matrix to allow encapsulation at a lower temperature and the impact of adding nutrients to the matrix were both assessed, independently and in combination, to determine the optimal encapsulating matrix for the survival of the three marine bacteria. 

## 2. Materials and Methods

To determine the impact of matrix composition on bacterial viability during encapsulation, three representative marine bacteria (*M. polaris, P. aquimaris,* and *B. licheniformis*), isolated from intertidal marine sediments in Prince Edward Island, Canada, were prepared and encapsulated based on our previous methods [11]. Briefly, each bacterial strain was inoculated in 5 mL of marine broth (BD Difco™, Fischer Scientific, Waltham, MA, USA) and incubated at room temperature on a shaker at 200 rpm for 48 h. Following inoculation, the cell density of each culture (an approximation of concentration) was determined using optical density at 600 nm measured with a NanoDrop^®^ ND-1000 Spectrophotometer (ThermoFisher, Waltham, MA, USA), according to previous protocols [11]. As a cell concentration of 7.64 × 10^6^ cells/mL was required for optimal single-cell encapsulation (following Poisson distribution), this concentration of each strain was centrifuged at 4500× *g* for 5 min to obtain a cell pellet [11]. The obtained bacterial pellets were suspended in each representative matrix, as described in Table 2. Ultrapure agarose (Sigma Aldrich, Oakville, ON, Canada) was maintained at 45 °C during encapsulation, while the agarose with low gelling temperature (Sigma Aldrich, USA) was maintained at 35 °C, representing a significant temperature difference. All species were encapsulated in each matrix in triplicate. 

Marine bacteria were encapsulated according to our previous methods [11]. In summary, bacteria were encapsulated within 80 ± 20 µm microbeads using a dispersed phase consisting of the agarose bacterial suspension at a flow rate of 5 mL/h and a continuous phase consisting of mineral oil (Sigma Aldrich, Oakville, ON, Canada), containing 4% *v*/*v* Span^®^ 80 nonionic surfactant (Sigma Aldrich, Oakville, ON, Canada) at a flow rate of 110 mL/h. To prevent premature gelation of the agarose, the reservoir, tubing, and microfluidic chip were heated [11]. Mineral oil was removed from the samples by sequentially straining the microbead suspensions over 60 µm and 100 µm cell strainers (pluriSelect Life Science, Leipzig, SN, Germany). Diluted marine broth (1:10 dilution) was used for straining the microbeads. Microbeads were also prepared without bacterial inoculation as a control. As a comparison, cell pellets were also resuspended in diluted marine broth using a 1:1000 dilution factor, which equates to the same concentration of cells per volume as that used in the encapsulated samples following the encapsulation protocol. 

The number of viable bacteria in each sample was then assessed by colony counts. Colony counts were performed by spreading 100 µL aliquots of each sample onto marine agar plates (*n* = 3). All plates were incubated at room temperature (20 °C) for five days prior to counting the number of colonies observed on each plate. To further assess the effect of a lower encapsulating temperature on bacterial viability, triplicate samples of each representative species prior to encapsulation were heated to 35 °C in a water bath for one hour (representative of the time it takes for encapsulation). Control samples, in triplicate, for each species were maintained at room temperature. The viability of each species at both temperatures was determined using colony counts and the PrestoBlue^®^ assay (ThermoFisher, Waltham, MA, USA). The PrestoBlue^®^ assay was performed, according to previous methods, providing an indication of metabolic activity with the reduction of resazurin to resorufin [11]. Results were indicated in terms of relative fluorescent units (RFUs), and high fluorescent intensities correspond to increased metabolic activity and, consequently, more viable cells [11]. 

All statistical analyses were performed using Prism 8 (GraphPad, La Jolla, CA, USA). Statistical significance was determined using unpaired, two-tailed *t*-tests and the Holm-Sidak method of correction for *p*-values, with an alpha of 0.05. Each dataset was analyzed individually without assuming a consistent standard deviation. 

## 3. Results and Discussion

**Figure 1:**
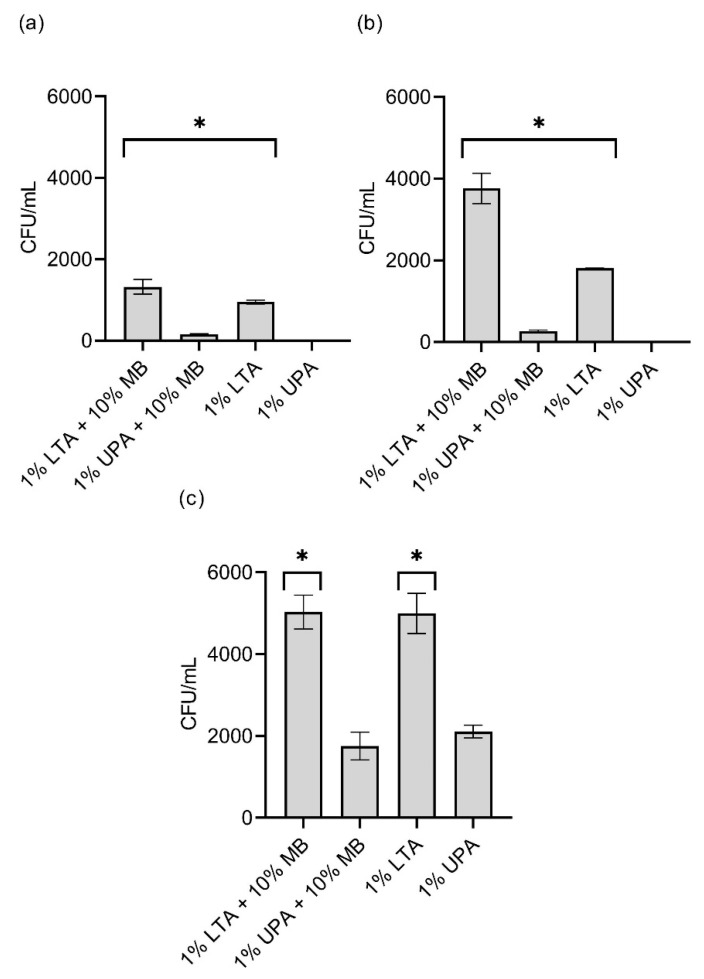
Effect of matrix on encapsulation. Viability after encapsulation of three representative marine sediment bacteria: (**a**) *M. polaris*, (**b**) *P. aquimaris*, and (**c**) *B. licheniformis*, using four matrices assessed via colony counts (*n* = 3) measured by colony-forming units per volume (CFL/mL). Error bars reflect standard error, and * denotes significance of *p* < 0.05 between modified matrix and 1% UPA.

Based upon an assessment of the various matrix compositions and conditions, it was determined that, as expected, the greatest impact on bacterial viability and abundance for all species was temperature. The use of two different types of agaroses requiring different temperature conditions assessed the impact of temperature and an altered agarose composition on viability. The effect of nutrient content was determined by supplementing the agarose matrices with diluted marine broth. More specifically, when assessing the impact of encapsulating matrix on *M. polaris*, it was determined that there was a significant increase in viability when using a modified agarose matrix (low gelling temperature or addition of marine broth), compared with the use of 1% ultrapure agarose (Figure 1a) [11]. The use of low-gelling-temperature agarose had a greater impact on viability than the addition of nutrients for this species, as there was no significant difference in cell viability between the sample with and without nutrients when using low-gelling-temperature agarose (Figure 1a). Consequently, it was determined that, as long as this species is encapsulated at a lowered temperature, the addition of nutrients has a negligible effect. However, when encapsulated at a higher temperature, the addition of nutrients benefits the growth of the decreased number of cells that survive encapsulation. This observation is consistent with the growth requirements of *M. Polaris*, as it is known to be adapted to cold-water conditions and is tolerant of oligotrophic conditions; thus, the use of low-gelling-temperature agarose is assumed to have a greater significance on improved viability, compared with the addition of nutrients [18]. 

When assessing the impact of encapsulating matrix on *P. aquimaris*, a similar trend was observed (Figure 1b). While there was a significant difference observed between all combinations of encapsulating matrices, it appeared that the lower encapsulating temperature again had a more significant effect on viability than nutrient addition. Both *M. polaris* and *P. aquimaris* have an optimal growth temperature below 35 °C; thus, heating these species to any temperature above 35 °C is assumed to negatively affect viability. This further indicates that the use of low-gelling-temperature agarose and the corresponding lower encapsulating temperature allows a greater proportion of psychrotolerant marine bacteria to survive the encapsulation process [18].

With an optimal growth temperature of 50 °C and an ability to survive under harsher conditions, *B. licheniformis* has been shown to tolerate a variety of encapsulation matrices and conditions [19]. Thus, as may be predicted, the viability of *B. licheniformis* was less affected by the use of 1% ultrapure agarose (1% UPA) than *M. polaris* and *P. aquimaris.* This is likely due to the thermophilic nature of B. licheniformis [11]. Despite the well-recognized thermal tolerance of B. licheniformis, the use of low-gelling-temperature agarose resulted in approximately 2.5-fold higher viability, compared with 1% UPA. As with *M. polaris* and *P. aquimaris*, the addition of dilute marine broth had a negligible effect on the viability of *B. licheniformis* (Figure 1c). This may be due to the fact that a relatively small amount of nutrients were added to the matrix, and this species is known to benefit from a eutrophic environment [20]. As a result, the addition of a greater concentration of nutrients to the matrix would likely have had a greater effect on the viability of this species. 

**Figure 2:**
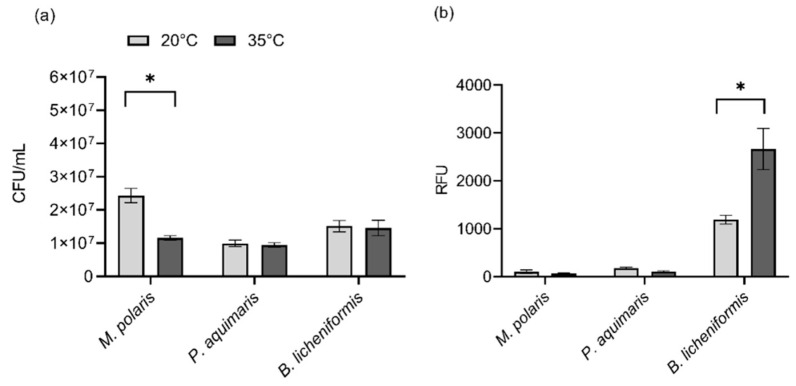
Effect of temperature (35 °C) on bacterial viability. Representative marine sediment bacteria (*M. polaris*, *P. aquimaris*, and *B. licheniformis*) were incubated at room temperature (20 °C) or subjected to heat (35 °C) for 1 h. Cell viability was determined using (**a**) colony counts after 5 days of growth measured with colony-forming units per volume (CFL/mL) and (**b**) the PrestoBlue^®^ assay measured with relative fluorescent units (RFUs). Error bars reflect standard error, and * denotes significance of *p* < 0.05.

Due to the observation that the use of low-gelling-temperature agarose and the corresponding lower encapsulating temperature (35 °C) appeared to have the most significant impact on bacterial viability for the three test species (Figure 1), the effect of temperature alone on viability was also assessed via comparison to room temperature (20 °C). Based upon viability assessment using colony counts, there was no significant difference in viability between 35 °C and room temperature for *P. aquimaris* (*t*(4) = 0.44, *p* = 0.8988) and *B. licheniformis* (*t*(4) = 0.19, *p* = 0.8988) while the viability of *M. polaris* was significantly reduced at 35 °C compared to room temperature (*t*(4) = 5.61, *p* = 0.0148) (Figure 2a). While *M. polaris* showed decreased viability at 35 °C, compared with room temperature, viability was improved significantly in comparison to previous studies examining the impact of a temperature of 45 °C on this species, which was required for encapsulation using ultrapure agarose rather than low-gelling-temperature agarose [11]. When assessing viability using the PrestoBlue^®^ assay, there was no significant difference for *M. polaris* (*t*(6) = 0.95, *p* = 0.3779) and *P. aquimaris* (*t*(6) = 2.89, *p* = 0.0549) between the two temperatures, indicating that the metabolic activity was not adversely impacted by a temperature of 35 °C (Figure 2b). On the other hand, there was a significant increase in the metabolic activity of *B. licheniformis* (*t*(6) = 3.36, *p* = 0.0449) at the increased temperature of 35 °C, compared with room temperature. Thus, even bacteria that adapted to cooler conditions, such as *M. polaris* and *P. aquimaris*, did not appear to have a lowered viability, indicated by a measure of metabolic activity, although growth on an agarose plate was inhibited for *M. polaris*. Consequently, the heat required for encapsulation using low-gelling-temperature agarose was shown to have a neutral or even positive impact on bacterial viability.

## 4. Conclusions

Upon assessing two agarose-based encapsulating matrices—two nutrient compositions and two encapsulation temperatures—it was determined that the temperature had a more significant impact on bacterial viability than varying nutrient composition. Thus, modification of our previous cultivation method to use a lower encapsulating temperature by using low-gelling-temperature agarose may improve the recovery of a greater diversity of species from marine habitats, thus facilitating the cultivation of new bacterial taxa [11]. This study further demonstrated that the agarose composition used for microencapsulation of environmental bacteria can be easily modified based on the environment of interest to maximize the viability and recovery of extant bacterial inhabitants [11]. Future experiments will be aimed at modifying the matrix to further select for specific bacterial taxa. Additionally, a combination of matrix compositions may be used to improve the cultivability of bacteria within a sample, as different bacteria present within the same sample may require slightly different growth conditions.

## Figures and Tables

**Table 1:** Characteristics of three bacterial species isolated from marine habitats in Prince Edward Island, Canada [16,17,18].

Species	*Marinomonas polaris* (RKSB-7)	*Psychrobacter aquimaris* (RKSB-1A)	*Bacillus licheniformis* (RKHZ-116)
Phylum	*Proteobacteria*	*Proteobacteria*	*Firmicutes*
Class	*Gammaproteobacteria*	*Gammaproteobacteria*	*Bacilli*
Order	*Oceanospirillales*	*Halobacteriales* or *Pseudomonadales*	*Bacillales*
Family	*Oceanospirillaceae*	*Moraxellaceae*	*Bacillaceae*
Cell shape	Rod	Coccus	Rod
Motility	Motile	No	Motile
Salt Requirement	Growth: 0–11%	Growth: 0–12%	Growth: unknown
	Optimum: 5%	Optimum: 2.5%	Optimum: unknown
Oxygen tolerance	Aerobe	Facultative anaerobe	Aerobe
Spore formation	No	No	Yes
Temperature Range	Psychrotolerant	Psychrotolerant	Mesophilic
pH	Growth: 6–10	Growth: 5–7.5	Growth: unknown
	Optimum: 7	Optimum: 7	Optimum: unknown
Location	Malpeque Bay, PE, Canada	Malpeque Bay, PE, Canada	Brackley, PE, Canada
	46.620818, −63.910324	46.620818, −63.910324	46.430210, −63.197933
Environments isolated from	Intertidal marine sediment	Intertidal marine sediment	Decayed feather in seawater
Biosafety level	1	1	1

**Table 2:** Composition of matrices.

Sample Name	Agarose Composition	Nutrient Composition
1% UPA	1% *w*/*v* ultrapure agarose	none
1% LTA	1% *w*/*v* low-gelling-temperature agarose	none
1% UPA + 10% MB	1% *w*/*v* ultrapure agarose	10% *w*/*v* marine broth
1% LTA + 10% MB	1% *w*/*v* low-gelling-temperature agarose	10% *w*/*v* marine broth

## Data Availability

Data are available upon request.
